# Identification and Biological Characterizations of the Causal Agent of Leaf Spot Disease in *Pseudostellaria heterophylla*

**DOI:** 10.3390/plants15060883

**Published:** 2026-03-12

**Authors:** Yunbo Kuang, Qian Chen, Felix Abah, Jiyu Su, Yujin Yang, Qiyuan Yang, Zuyun Ye, Zonghua Wang, Meilian Chen, Hongli Hu

**Affiliations:** 1 The Engineering Technology Research Center of Characteristic Medicinal Plants of Fujian, College of Biological Science and Engineering, Ningde Normal University, Ningde 352100, China; kuangyb@ndnu.edu.cn (Y.K.);; 2 College of Life Science, Capital Normal University, Beijing 100048, China; 3 College of Plant Protection, Fujian Agriculture and Forestry University, Fuzhou 350002, China; fabah11@gmail.com (F.A.);; 4 Fuzhou Institute of Oceanography, College of Materials and Chemical Engineering, Minjiang University, Fuzhou 350108, China

**Keywords:** *Pseudostellaria heterophylla*, biological characterizations, *Sclerotiophoma versabilis*, leaf spot disease, multi-locus phylogenetic analyses

## Abstract

*Pseudostellaria heterophylla*, an important traditional medicinal plant in China, has suffered increasing yield and quality loss due to leaf spot disease in recent years. In this study, the causal agent was conclusively identified as *Sclerotiophoma versabilis* through detailed morphological characteristics and multi-locus phylogenetic analyses based on the internal transcribed spacer regions (ITS), the 28S large subunit of the nrDNA (LSU), RNA polymerase II (*rpb2*), and ß-tubulin (*tub2*) sequences. Pathogenicity tests fulfilled Koch’s postulates, thereby resolving previous taxonomic inconsistencies regarding this disease. The effects of environmental and nutritional factors on mycelial growth, conidial germination, and infection were systematically evaluated. Optimal mycelial growth occurred at 20–25 °C, pH 6–8, under continuous light. Optimal mycelial growth occurred at 20–25 °C, pH 6–8, under continuous light, while conidial germination was maximized at 20–25 °C and pH 6–7 under continuous light. Starch and glycine were identified as the most favorable carbon and nitrogen sources for the fungal mycelial growth, respectively. Infection assays indicated an incubation period of approximately 3 d and maximal disease development at moderate temperatures under low-light conditions, with 6 d-old cultures exhibiting the greatest infectivity. Microscopic observations revealed that *S. versabilis* penetrated host tissues directly or via stomata without forming specialized infection structures. These findings integrate taxonomic resolution with ecological and infection biology analyses, providing mechanistic insight into the environmental drivers of leaf spot epidemics and a scientific basis for disease-risk assessment and management in *P. heterophylla* production systems.

## 1. Introduction

*Pseudostellaria heterophylla* (Miq.) Pax is a geo-authentic medicinal herb extensively used in traditional Chinese medicine. Modern pharmacological studies have demonstrated that *P. heterophylla* contains diverse bioactive compounds, including polysaccharides, saponins, amino acids, cyclic peptides, and volatile oils. Its medicinal preparations exhibit multiple therapeutic properties, such as antitussive, anti-cancer and antioxidant activities, as well as alleviation of fatigue and intestinal inflammation [1,2,3,4,5,6].

However, the sustainable production of *P. heterophylla* has been seriously challenged by fungal diseases, including black spot caused by *Arcopilus aureus* [7], brown spot caused by *Stemphylium solani* [8], and damping-off caused by *Rhizoctonia solani* and *Alternaria alternata* [9]. Among these, leaf spot disease has emerged as one of the most destructive constraints to commercial cultivation, occurring widely across major planting regions in China [10,11,12]. Under continuous monoculture, disease incidence increases annually despite routine fungicide application. Prolonged reliance on chemical control has resulted in declining yields, reduced product quality, and growing environmental concerns, thereby posing serious challenges to sustainable production [11,13,14].

In response to these production challenges, modern plant disease management increasingly emphasizes early detection and accurate pathogen identification as prerequisites for integrated and evidence-based control. This requirement is particularly critical for medicinal and specialty crops, which often receive limited phytosanitary surveillance but are highly sensitive to disease-related quality deterioration. Recent advances in plant disease diagnostics further indicate that reliable disease management depends not only on precise taxonomic identification but also on comprehensive biological characterization, which together support disease-risk assessment, epidemiological forecasting, and targeted intervention strategies [15,16].

Although several studies have attempted to identify the causal agent of leaf spot disease in *P. heterophylla*, their conclusions have remained inconsistent. Early reports attributed the disease to *Phoma* sp. [17,18] or to *Phyllosticta commonsii* [10,19] based solely on morphological traits, while later work identified the pathogen as *Ascochyta versabilis* (=*Sclerotiophoma versabilis*) using the internal transcribed spacer regions (ITS)-based phylogenetic analysis together with limited cultural and conidial characteristics [11]. More recently, Yao et al. [14] re-identified it as *S. versabilis* using multi-locus phylogenetic analyses, including ITS, the 28S large subunit of the nrDNA (LSU), and ß-tubulin (*tub*). However, additional evidence from detailed cultural characteristics, micromorphological features or molecular phylogenetic analyses would further strengthen taxonomic resolution. Consequently, the etiology and epidemiology of leaf spot disease in *P. heterophylla* remain incompletely understood.

These inconsistent conclusions are closely related to the complex taxonomic history of phoma-like fungi. *Phoma* was historically regarded as one of the largest fungal genera, comprising more than 3000 subgenera (infrageneric taxa) [20]. Subsequently, the family *Didymellaceae* was established to accommodate *Phoma*, *Ascochyta*, *Didymella*, and several other related genera [21]. Recent taxonomic revisions within *Didymellaceae* have reshaped our understanding of phoma-like fungi, using multi-loci analyses combined with morphological differences to resolve species boundaries [22,23,24].

Notably, previous studies on *P. heterophylla* leaf spot disease have primarily focused on pathogen identification and fungicide screening [10,11,14,17,18,19]. Systematic investigations of infection biology, and physiological adaptation, and quantitative environmental thresholds have rarely been conducted. As a result, the mechanisms underlying disease development and environmental adaptation of *S. versabilis* in *P. heterophylla* plantations remain largely unclear. This lack of integrative biological and ecological information has hindered the development of predictive models and targeted disease management strategies.

Accurate identification and comprehensive biological characterization of the causal pathogen are therefore essential for advancing research on pathogenesis, epidemiology, and host–pathogen interactions, as well as for improving sustainable control practices. In this study, we present a systematic investigation integrating detailed morphological characterization, multi-locus phylogenetic analyses, physiological profiling, and infection cytology. Specifically, we aimed to (i) isolate and accurately identify the causal agent of leaf spot disease in *P. heterophylla*; (ii) determine the environmental conditions influencing vegetative growth, conidial germination, and infection; and (iii) elucidate the cytological processes of host penetration and colonization. Beyond taxonomic resolution, quantitative characterization of pathogen biology provides useful parameters for crop health monitoring and disease-risk forecasting, which is increasingly important for medicinal and specialty crops. Recent advances in field-deployable vision systems have demonstrated that deep learning and the Internet of Things-enabled frameworks can support disease detection, severity assessment, and crop loss estimation under field conditions [25,26], although practical deployment remains challenged by data variability and scalability [27]. Within this broader context, the environmental thresholds and infection-related traits defined here provide mechanistic inputs that can complement modern monitoring pipelines and guide integrated management decisions. By linking taxonomic, physiological, and cytological evidence, this work provides a biologically informed framework for disease-risk assessment, epidemiological forecasting, and the development of sustainable management strategies for this economically important medicinal crop.

## 2. Materials and Methods

### 2.1. Samples, Isolation and Pathogenicity Test of Pathogen

The leaves exhibiting typical leaf spot symptoms of *P. heterophylla* were collected from Zherong County (27°14′10″ N, 119°53′57″ E), Ningde City, Fujian Province, China, in late April 2017. Small tissue pieces (3 × 3 mm), cut from the leading edges of lesions, were surface–disinfected in 75% (*v*/*v*) ethanol for 10 s, followed by 1% (*v*/*v*) NaClO for 2–3 min, rinsed with sterile water for 3–5 times, and blotted dry with sterilized filter paper. The disinfected tissues were placed onto potato dextrose agar media [PDA, 40.1 g PDA powder in 1 L double-distilled water (ddH_2_O)] (Guangdong Huankai microbial and Tech. Co., Ltd., Guangzhou, China) and incubated at 25 °C under a 12 h light/12 h darkness (12 h L/12 h D) cycle for 3–5 d. If mycelia appeared around the leaf fragments, purification was carried out.

The pathogenicity of the representative isolate was determined according to Koch’s postulates. Both detached leaf and in vivo pathogenicity assays were performed. The detached-leaf assay was performed as a rapid preliminary screening of isolate aggressiveness using mycelial plugs, which are easy to handle and provide consistent inoculum. Briefly, 5 mm mycelial PDA plugs were inoculated onto pre-wounded detached leaves, with sterile PDA plugs as controls.

For in vivo pathogenicity confirmation, intact plantlets were inoculated by spraying a mycelial suspension, which better mimics natural infection. 3 d-old mycelia from the potato dextrose broth (PDB, 24 g PDB powder in 1 L ddH_2_O) (Guangdong Huankai microbial and Tech. Co., Ltd., Guangzhou, China) were collected and ground, then resuspended in ddH_2_O containing 0.02% (*v*/*v*) Tween 20, sprayed onto pre-injured intact plants, with sterile ddH_2_O as controls. Inoculated leaves/plantlets were then maintained at 25 °C, under high humidity for 7 d under a 12 h L/12 h D cycle.

### 2.2. Morphology

Morphological studies were conducted following the methods described in previous studies, with minor modifications [22,23,24]. The isolate was inoculated on 2% malt extract agar [MEA, 20 g maltose extract (Oxoid Ltd., Basingstoke, Hampshire, UK), 20 g agar dissolved in 1 L ddH_2_O, oatmeal agar (OA; Oatmeal 30 g, agar 20 g dissolved in 1 L ddH_2_O] and PDA at 25 °C under a 12 h L/12 h D photoperiod, and on water agar with in vitro detached leaves of *P. heterophylla* to induce sporulation. Colony diameters were measured after 7 d, and colony morphologies determined after 14 d of incubation. Micromorphological descriptions and measurements for 30 replicates of relevant features were carried out from mature pycnidia and conidia mounted in water. For conidiomatal pycnidia and pycnidial walls, measurements were taken from 5 to 10 samples. Observations were conducted with an Olympus DP80 light microscope (Olympus Corporation, Hachioji, Tokyo, Japan). Sections of pycnidia were prepared using a SLEE MEV freezing microtome (SLEE medical GmbH, Mainz, Germany) to study the anatomy of pycnidial walls.

### 2.3. DNA Isolation and PCR Amplification

Genomic DNA was extracted from fresh mycelia growing on PDA using the CTAB method [28]. Four loci, including LSU [29,30], ITS [31,32], *tub2* [33], and RNA polymerase II (*rpb2*) [34,35] were amplified and sequenced. PCR amplifications were performed in a reaction mixture consisting of 12.5 μL 2 × Taq PCR Mastermix [TIANGEN Biotech (Beijing) Co., Ltd., Beijing, China], 1 μL each of 10 μM primers, 1 μL of genomic DNA, adjusted to a final volume of 25 μL with ddH_2_O. The PCR primer pairs and amplification conditions are listed in Table 1. The PCR products were sequenced by Fuzhou Sunya Biotechnology Co., Ltd., Fuzhou, China). Novel sequences generated in this study were deposited in GenBank (http://www.ncbi.nlm.nih.gov, Table 2).

### 2.4. Molecular Phylogenetic Analyses

Phylogenetic analyses were performed following the protocol of Hou et al. [24]. Reference sequences from representative species of *Didymellaceae* were downloaded from Genbank and are listed in Table 2. Consensus sequences were obtained using MEGA v.11 software [36] and sequences for four individual loci (ITS, LSU, *rpb2* and *tub2*) were aligned using MAFFT v.7 [37]. A multi-locus sequence dataset was generated using SequenceMatrix v. 1.8 [38]. Phylogenetic analyses were carried out by both maximum likelihood and Bayesian Inference methods using PhyloSuite (v1.2.2) [39], with *Pleiochaeta setosa* (CBS 118.25) serving as the outgroup taxon.

### 2.5. Effects of Cultural Conditions on the Mycelia Growth

Mycelial plugs of isolate KC1 were inoculated on PDA and incubated at 25 °C, under a 12 h L/12 h D cycle for 6 d. Then plugs of 5 mm in diameter were excised from the actively growing edges of the 6 d-old culture. All experiments were performed with three independent biological replicates, each consisting of five technical replicates per treatment. All data were processed using IBM SPSS statistics 19.0 and GraphPad Prism 5.0.

#### 2.5.1. Effect of Culture Medium on Mycelial Growth

The prepared mycelial plugs were inoculated onto complete medium (CM; 6 g yeast extract powder, 6 g hydrolyzed casein, 10 g sucrose, 20 g agar dissolved in 1 L ddH_2_O), mulberry agar (MA; 33 g dried mulberry leaves, 10 g sucrose, 20 g agar dissolved in 1 L ddH_2_O and prune agar (PA; 40 mL prune juice, 2.5 g lactose, 2.5 g sucrose, 1 g yeast extract, 20 g agar dissolved in 1 L ddH_2_O). Then the plates were placed under a 12 h L/12 h D cycle at 25 °C. The colony diameters were measured after 7 d, and colony morphologies determined after 14 d of incubation.

#### 2.5.2. Effects of Temperature and Light Regime on Mycelial Growth

For temperature tests, PDA plates inoculated with 5 mm mycelial plugs were incubated at 5 °C, 10 °C, 15 °C, 20 °C, 23 °C, 25 °C and 30 °C in the dark. For light tests, PDA plates were placed under three light regimes: continuous light, a 12 h L/12 h D cycle, and continuous darkness at 25 °C. Colony diameters were measured after 7 d.

#### 2.5.3. Effect of pH on Mycelial Growth

PDB with different pH values (3, 4, 5, 6, 7, 8, 9, 10, 11, 12) were prepared with 0.1 M HCl or NaOH. Glucose solution was sterilized by filtration first and then was added to the sterile media. Fifteen mycelial plugs were transferred into 200 mL PDB in a 500 mL conical flask. The cultures were incubated at 25 °C in a rotary shaker, at 110 rpm for 3 d. Then, mycelia were filtered from the liquid media, washed twice with sterile ddH_2_O, and dried with sterile filter papers, then transferred to 50 mL centrifuge tubes to be fully dried at 60 °C for 12 h. Allow it to cool for 30 min at room temperature in the desiccator and then weight it until a constant weight (successive weightings agree to within ±0.0002 g) was achieved.

#### 2.5.4. Effects of Carbon and Nitrogen Sources on Mycelial Growth

To evaluate nutritional requirements, the influence of carbon and nitrogen sources on mycelial growth was assessed using water agar (WA) as the minimal medium. For carbon treatments, the carbon source was replaced individually with glucose, sucrose, starch, lactose, maltose, mannose, sorbose, or fructose. For nitrogen treatments, the nitrogen source was replaced with glycine, aspartate, peptone, histidine, proline, sodium nitrate (NaNO_3_), glutamic acid, valine, cysteine, or urea. Each medium was inoculated with a 5 mm mycelial plug and incubated at 25 °C in the dark. Colony diameters were recorded after 7 d.

The appropriate concentration for the carbon and nitrogen source in the subsequent experiments was selected respectively according to the principle of diminishing marginal returns. Mycelial plugs were inoculated on WA containing sucrose at different concentrations (10 g/L, 20 g/L, 30 g/L, 40 g/L, 50 g/L and 60 g/L) or containing proline at different concentrations (2.5 g/L, 5 g/L, 10 g/L, 15 g/L, 20 g/L, 25 g/L) and cultured at 25 °C in the dark.

#### 2.5.5. Determination of Lethal Temperature

The mycelial plugs were placed in 1.5 mL centrifuge tubes containing 1 mL of ddH_2_O and incubated in a thermostatic water bath at 40 °C, 45 °C, 50 °C, 55 °C, 60 °C, 65 °C, 70 °C and 75 °C for 10 min. The tubes were then immediately transferred to a 25 °C water bath for cooling. After treatment, the mycelial plugs were removed, excess water was absorbed with sterile filter paper, and the plugs were transferred onto PDA plates. The plates were incubated at 25 °C in the dark, and mycelia growth was determined after 7 d.

### 2.6. Growth Assays on Hydrophobic Slides (PHOB-S), Hydrophilic Slides (PHIL-S) and Onion Epidermis

Aliquots (20 μL) of conidial suspensions [(2–3) × 10^4^ spores/mL] were placed onto PHOB-S [GelBond^®^ Film Sheets (Lonza Rockland, Inc., Rockland, ME, USA)], PHIL-S [Fisherbrand microscope cover glass (Thermo Fisher Scientific, Waltham, MA, USA)] and onion epidermis, and incubated under humid conditions at 25 °C in the dark for 4 h, 6 h, 8 h, 12 h, 24 and 48 h. Germination was examined using a Olympus DP80 light microscope (Olympus Corporation, Hachioji, Tokyo, Japan). Germination rates on PHOB-S and PHIL-S were measured after 4 h, 6 h and 8 h. For each replicate, 100 spores were examined microscopically. All experiments were performed with three independent biological replicates, each consisting of three technical replicates per treatment.

### 2.7. Conidial Germination Assays

The pathogenic fungus was able to form pycnidia on artificial culture media, such as CM, MA, PA, MEA, OA and PDA. However, no conidia were produced on these media. Therefore, mycelial plugs were inoculated onto WA supplemented with leaves of in vitro-grown *P. heterophylla* plantlets and cultured at 25 °C under a 12 h L/12 h D photoperiod for 1 w. Conidia were collected by washing the leaves with sterile ddH_2_O. All experiments were performed with three independent biological replicates, each consisting of three technical replicates per treatment.

#### 2.7.1. Effects of Temperatures and Light Regimes on Conidial Germination

Aliquots (20 μL) of conidial suspension (1 × 10^5^ spores/mL) were placed on the microscope slides and incubated under humid conditions at 15 °C, 20 °C, 25 °C, 30 °C and 35 °C in the dark. For photoperiod treatments, conidia were incubated at 25 °C under continuous light, a 12 h L/12 h D cycle, and continuous darkness. Conidial germination rates were measured after 4 h, 6 h and 8 h. For each replicate, 100 spores were examined microscopically.

#### 2.7.2. Effect of pH on Conidial Germination

Aliquots (20 μL; 1 × 10^5^ spores/mL) of conidial suspension were prepared in 0.05 M glycine buffer adjusted to pH 3, 4, 5, 6, 7, 8, 9 and 10. Drops were placed onto the microscope slides and incubated under humid conditions at 25 °C under continuous light. Conidial germination rates were recorded 6 h post-inoculation (hpi). For each replicate, 100 spores were examined microscopically.

### 2.8. Effects of Environmental Factors and the Mycelial Age on Infection

To evaluate the effects of temperature and photoperiod on infection, detached pre-wounded leaves of the *P. heterophylla* plantlets were inoculated with 6 d-old mycelial plugs or 30 μL conidial suspensions (1 × 10^6^ spores/mL). Control leaves received either 5 mm PDA plugs or 30 μL of sterile ddH_2_O. The inoculated leaves were then incubated at 15 °C, 20 °C, 25 °C, 30 °C under a 12 h L/12 h D photoperiod, or at 25 °C under continuous light, a 12 h L/12 h D photoperiod, or continuous darkness. To assess the influence of mycelial age, plugs of 3, 6, 9, 12, and 15 d-old cultures were inoculated onto pre-injured leaves and incubated at 25 °C under a 12 h L/12 h D cycle.

The infection process was monitored every 12 h, and the incubation period was recorded. Photographs were taken, and lesion areas were quantified by ImageJ.JS (web version of ImageJ; https://ij.imjoy.io/, accessed on 12 November 2025) after 7 d. Digital images were captured under standardized conditions. Lesion boundaries were manually delineated in ImageJ according to predefined criteria to ensure consistency across samples. All measurements were performed in a standardized manner to minimize observer bias. All experiments were performed with three independent biological replicates, each consisting of three technical replicates per treatment.

### 2.9. Cytological Observation of the Infection Process

Conidial suspensions were adjusted to (2–3) ×10^4^ spores/mL in 0.02% Tween solution and applied to onion epidermis and leaves of *P. heterophylla* following the methods of Viaud et al. [40] and Yang et al. [41], with minor modifications. Samples were incubated in 12-well plates at 25 °C under a 12 h L/12 h D photoperiod. Infection-related morphogenesis on onion epidermis was observed at 6 h, 12 h, 24 h, 36 h and 48 h using a light microscope (Olympus, Tokyo, Japan).

Based on observations from the onion penetration assay, infection events on *P. heterophylla* leaves were further examined. Aliquots (30 μL) of conidial suspension were inoculated onto the lower epidermis of the detached leaves and incubated under humid condition at 25 °C with a 12 h L/12 h D photoperiod. At 6, 12, 24, 36, 48, 60 and 72 hpi, leaf epidermal tissues were peeled, stained with 5% lactophenol cotton blue (Shanghai Yuanye Bio-Technology Co., Ltd., Shanghai, China), and examined using a Leica DM3000 light microscope (Leica Microsystems, Wetzlar, Germany). Conidial germination, penetration, invasion, colonization, and in-host hyphal expansion were recorded.

## 3. Results

### 3.1. The Causal Agent of Leaf Spot Disease in P. heterophylla Was Isolated and Identified as S. versabilis

**Figure 1:**
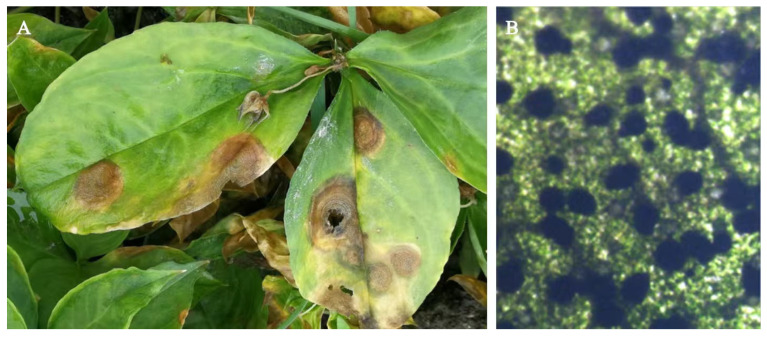
Symptoms of leaf spot disease on *P. heterophylla* in the field. (**A**). Typical necrotic symptoms on infected leaves. (**B**). Black pycnidia arranged in concentric rings within lesions.

**Figure 2:**
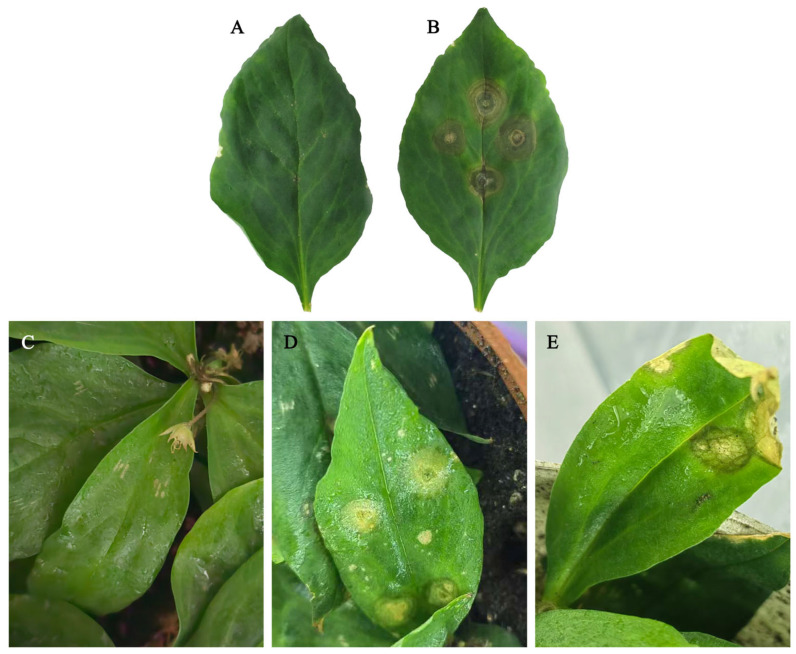
Pathogenicity of *S. versabilis* KC1 on *P. heterophylla*. (**A**,**B**) Lesion development on detached leaves. (**A**). Control inoculated with sterile PDA plugs; (**B**). Inoculated with isolate KC1. (**C**–**E**) Symptom development on intact plants ((**C**). Control inoculated with sterile ddH_2_O; (**D**). Symptoms at 4 d post inoculation; (**E**). Symptoms at 7 d post inoculation).

Naturally infected *P. heterophylla* leaves exhibited brown to dark brown, elliptical to circular necrotic lesions with yellow margins, accompanied by black pycnidia arranged in concentric rings on the lesions. In severe cases, the leaves withered, and the whole plant could die (Figure 1). Symptomatic leaf samples were collected for pathogen isolation and purification. Seven isolates (KC1–KC7) were obtained, all of which exhibited highly similar colony characteristics on PDA and comparable microscopic features. Based on this consistency, isolate KC1 was selected as a representative strain for pathogenicity assays, morphological observation and phylogenetic analyses. Pathogenicity tests demonstrated that KC1 successfully infected both detached leaves and intact plants of *P*. *heterophylla*, whereas no symptoms were observed on the control plants (Figure 2). The same fungus was re-isolated from inoculated lesions and exhibited morphological characteristics identical to those of the original cultures, thereby fulfilling Koch’s postulates.

**Figure 3:**
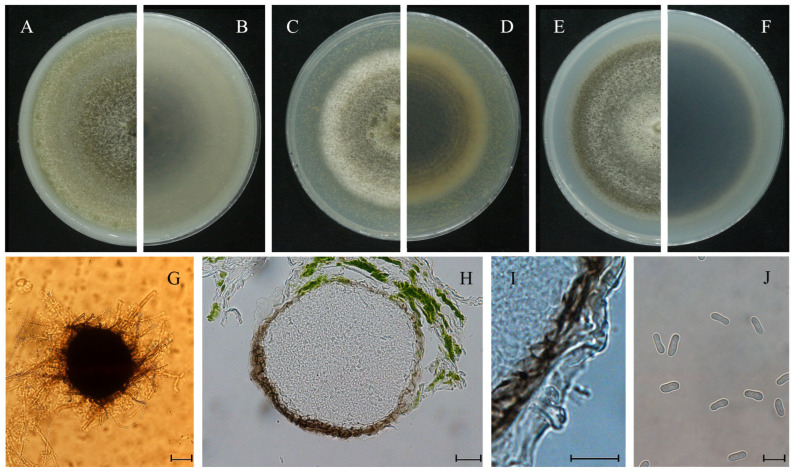
Colonies on media and microscopic characteristics of *S. versabilis*. (**A**,**B**) Colony on OA (front and reverse). (**C**,**D**) Colony on MEA (front and reverse). (**E**,**F**) Colony on PDA (front and reverse). (**G**) Pycnidium. (**H**) Section of pycnidium. (**I**) Section of pycnidial wall. (**J**) Conidia. Scale bars: (**G**) = 200 μm; (**H**) = 20 μm; (**I**,**J**) = 10 μm.

Morphological characteristics were examined on OA, MEA and PDA media. On OA, colonies attained a diameter of 62 mm and showed regular margins with sparse aerial mycelia. The colony was olivaceous at the center and pale grayish olivaceous toward the margin, with gray pycnidia arranged in concentric rings, whereas the reverse was olivaceous centrally and gray toward the margin (Figure 3A,B). On MEA, colonies reached 37 mm in diameter, with regular margins and felty aerial mycelia. The colony was dark gray at the center and pale gray toward the margin, with abundant pycnidia in concentric rings, and the reverse was concolorous with the front (Figure 3C,D). On PDA, colonies reached 45 mm in diameter and displayed regular margins with white, sparse aerial mycelia. The colony was olivaceous, with a white center and gray marginal zones, and produced abundant pycnidia in concentric rings, while the reverse was dark brown centrally and gray toward the margin (Figure 3E,F). On water agar with detached leaves of in vitro *P. heterophylla*, pycnidia were mostly 240–320 μm in diameter, with a dark periphery which disintegrated afterwards (Figure 3G–I). Conidia were subcylindrical, hyaline, smooth and thin-walled, mostly aseptate and sometimes uniseptate, eguttulate, and measured (6.2–) 8.0–11.1 (–12.7) × (2.6–) 3.0–4.7 (–5.4) μm (Figure 3J).

**Figure 4:**
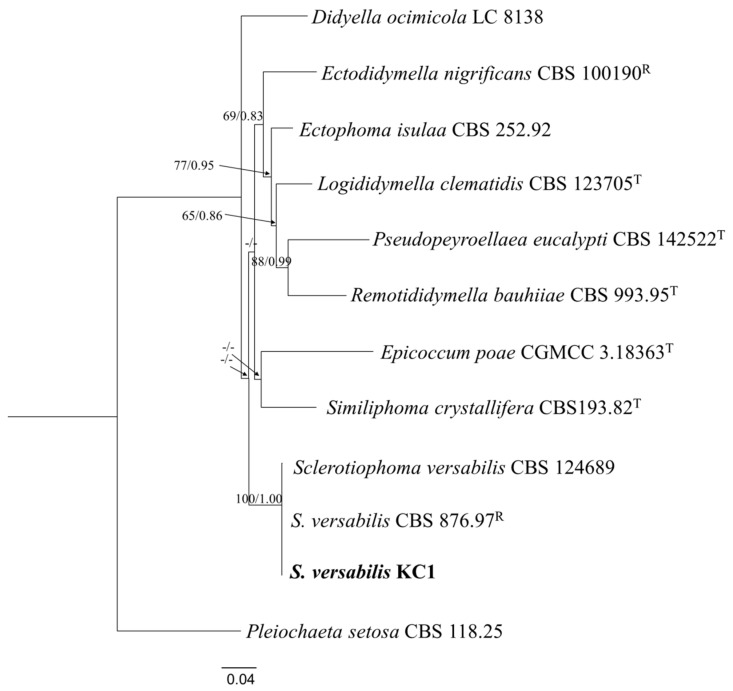
Maximum likelihood (ML) tree constructed by PhyloSuite (v1.2.2) with the combined *ITS*, *LSU*, *rpb2* and *tub2* sequences. *Pleiochaeta setosa* (CBS 118.25) served as the outgroup taxon. Numbers in front of slash stand for bootstrap values, and numbers behind the slash stand for the Bayesian posterior probabilities. “-” indicates that the given branches were supported less than 50% by maximum likelihood bootstrap or 0.80 by Bayesian analyses. The sequence obtained in this study was in bold. The scale bar represents the expected number of changes per site.

Morphological observations indicated that the isolate KC1 belonged to a *Phoma*-like fungus within the family of *Didymellaceae* [24]. Consistently, multi-locus phylogenetic analyses demonstrated that KC1 clustered tightly with reference strains of *S. versabilis* (strain CBS 124689 and CBS 876.97 R) with strong bootstrap support (Figure 4).

Based on the concordance between morphological characteristics and phylogenetic evidence, the causal agent of leaf spot in *P. heterophylla* was conclusively identified as *S. versabilis*.

### 3.2. Effects of Culture Conditions on the Vegetative Growth of S. versabilis

#### 3.2.1. Medium

**Figure 5:**
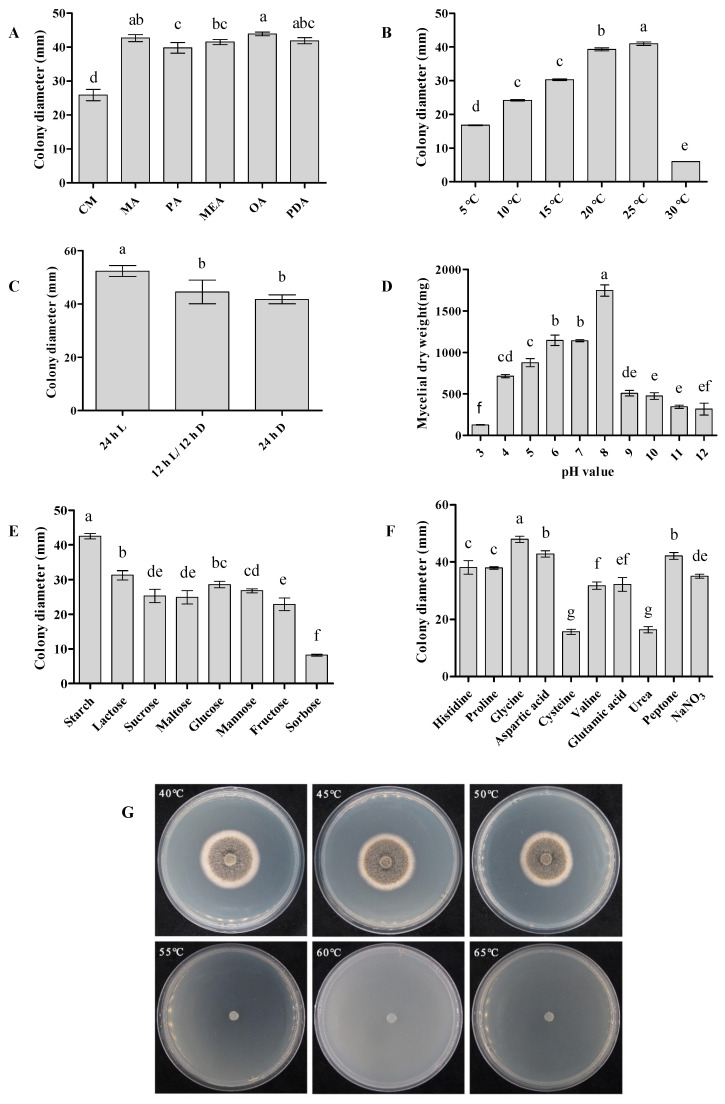
Effects of cultural conditions on *S. versabilis*. (**A**) Culture medium. (**B**) Temperature. (**C**) Light regime. (**D**) pH. (**E**) Carbon source. (**F**) Nitrogen source. (**G**) Lethal temperature determination. Data represent means ± standard error (SE) of three independent biological experiments, each with five technical replicates. Different lowercase letters indicate significant difference (*p* < 0.05) as determined by one-way ANOVA followed by Tukey’s multiple comparison test in SPSS 19.0. 24 h L = continuous light; 12 h L/12 h D = 12 h light/12 h darkness; 24 h D = continuous darkness.

Colony growth varied significantly among the six tested media. The largest colonies were obtained on OA (43.9 mm in diameter), whereas growth on CM was markedly reduced (25.9 mm) and significantly lower than that on all other media (*p* < 0.05). Colonies grown on OA, MA and PDA exhibited significantly greater expansion than those on CM, PA, and MEA (*p* < 0.05) (Figure 5A).

Across all media, colonies were olive green to brown and produced abundant pycnidia arranged in concentric rings, accompanied by felty aerial mycelia. In contrast, colonies on CM developed sparse aerial mycelia (Supplementary Figure S1).

#### 3.2.2. Temperature and Light

Mycelial growth increased progressively from 5 °C to 25 °C. Robust growth occurred at 20–25 °C, with colony diameters peaking at 41 mm at 25 °C, which was significantly greater than those at all other tested temperatures (*p* < 0.05). In contrast, temperatures above 25 °C markedly inhibited mycelial development (Figure 5B). Colony morphology also exhibited pronounced temperature-dependent variation. At 5–15 °C, colonies were compact with poorly developed aerial mycelia. In contrast, aerial mycelial development was markedly enhanced at 20–25 °C, whereas colonies grown at 30 °C displayed wrinkled surfaces, reduced aerial mycelia and sparse mycelial coverage (Supplementary Figure S2).

Light conditions significantly affected both colony growth and morphology. Continuous illumination promoted maximal growth (48.3 mm), which was significantly greater than that observed under a 12 h L/12 h D cycle or complete darkness (*p* < 0.05) (Figure 5C). Under continuous light, colonies were brown at the center and white toward the margin, producing dense, thick, felt-like aerial mycelia. Under a 12 h L/12 h D cycle, aerial mycelia were comparatively sparse, with distinct pycnidia forming in concentric rings. In continuous darkness, colonies were olive brown at the center and gray toward the margin, with sparse aerial mycelia (Supplementary Figure S3).

#### 3.2.3. pH

Mycelial biomass differed significantly across the tested pH range. Growth was maximal at pH 8 (1746 mg) (*p* < 0.05) and remained robust between pH 6 and 8. In contrast, pH values above 8 or below 6 markedly inhibited mycelial development (Figure 5D), indicating a preference for near-neutral to slightly alkaline environments.

#### 3.2.4. Carbon and Nitrogen Sources

Based on preliminary concentration screening, 10 g·L^−1^ and 2.5 g·L^−1^ were selected as optimal concentrations for evaluating carbon and nitrogen sources, respectively (Supplementary Figure S4A,B).

Among the tested carbon sources, starch supported significantly larger colonies (41.8 mm) and promoted dense mycelial growth, whereas sorbose resulted in significantly smaller colonies (8.2 mm) (*p* < 0.05). Lactose and glucose also supported substantial development (Figure 5E; Supplementary Figure S4C). Overall, complex carbohydrates and readily utilizable sugars favored vegetative growth.

For nitrogen sources, glycine supported the greatest colony expansion (47.9 mm), followed by aspartate and peptone, whereas cysteine (15.6 mm) and urea (16.4 mm) resulted in significantly reduced growth (*p* < 0.05) (Figure 5F; Supplementary Figure S4D). These results indicate that *S. versabilis* can utilize diverse nitrogen substrates but exhibits clear preferences for specific organic nitrogen sources.

Morphological observations further revealed that colonies grown on carbon-amended media consisted predominantly of vegetative mycelia, with limited aerial hyphae. In contrast, nitrogen supplementation generally promoted denser colony development, with prominent aerial hyphae observed on media containing peptone, proline, aspartate, and NaNO_3_ (Supplementary Figure S4C,D).

#### 3.2.5. Lethal Temperature

Mycelia survive and resumed growth following exposure to 40–50 °C, whereas no growth was observed at 55 °C, indicating that 55 °C represents the lethal temperature threshold for *S. versabilis* under the tested conditions (Figure 5G).

### 3.3. Characterization of Conidial Germination of S. versabilis

**Figure 6:**
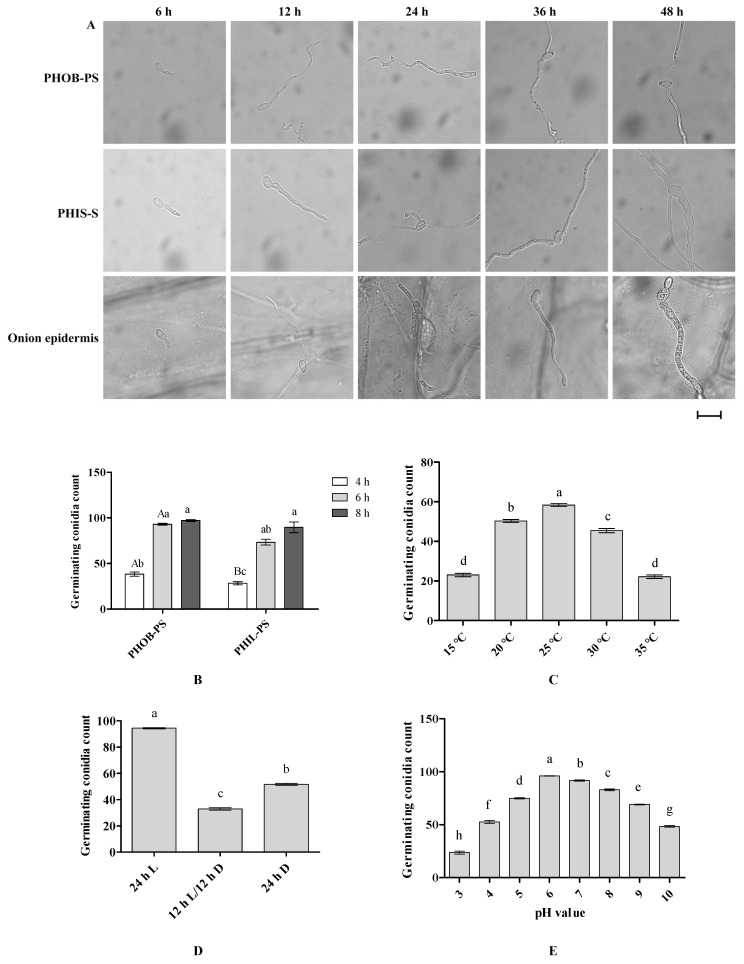
Characterization of conidial germination of *S. versabilis*. (**A**) Time-course images of conidia germination on hydrophobic slides (PHOB-S), hydrophilic slides (PHIL-S) and onion epidermis. Scale bar = 20 μm. (**B**) Conidial germination on PHOB-S and PHIL-S. Data were analyzed by two-way ANOVA followed by Tukey’s multiple comparison test using SPSS 19.0. Different uppercase letters indicate significant differences between surfaces at the same time point, while different lowercase letters indicate significant differences among time points within the same treatment (*p* < 0.05). (**C**–**E**) Effects of temperature, light regime and pH on conidial germination, respectively. Different lowercase letters indicate significant differences (*p* < 0.05) as determined by one-way ANOVA followed by Tukey’s multiple comparison test using SPSS 19.0. Data represent means ± SE of three independent biological experiments, each with three technical replicates. 24 h L = continuous light; 12 h L/12 h D = 12 h light/12 h darkness; 24 h D = continuous darkness.

Conidia germinated readily on PHOB-S, PHIL-S and onion epidermis, typically producing one to two germ tubes that elongated into hyphae. No specialized infection structures were observed. By 48 hpi, conidia and the adjacent hyphal cells were visibly swollen on PHIL-S and onion epidermis, whereas no such enlargement occurred on PHOB-S (Figure 6A). Surface properties significantly affected germination. Germination rates were significantly higher on PHOB-S than on PHIL-S at 4 hpi and 6 hpi, reaching 97.0% at 8 hpi (Figure 6B), indicating that PHOB-S promotes germination.

Temperature strongly influenced germination efficiency. Relatively high germination rates occurred at 20–25 °C, with a peak rate of 95.1% at 25 °C after 8 h (*p* < 0.05), whereas germination was markedly reduced at 30–35 °C (Figure 6C). Light conditions also affected germination. At 8 h, continuous light resulted in the highest germination rate (97.1%), significantly exceeding that observed under a 12 h L/12 h D cycle or complete darkness (*p* < 0.05) (Figure 6D). Germination varied with pH, reaching a maximum of 96.0% at pH 6, followed by pH7, both of which were significantly higher than those observed at other tested pH values (*p* < 0.05) (Figure 6E).

Overall, conidia germination was favored on PHOB-S, at moderate temperatures (20–25 °C) and weakly acidic to neutral conditions (pH 6–7), with particularly high rates under continuous light.

### 3.4. Characterization of the Infection Conditions of S. versabilis

**Figure 7:**
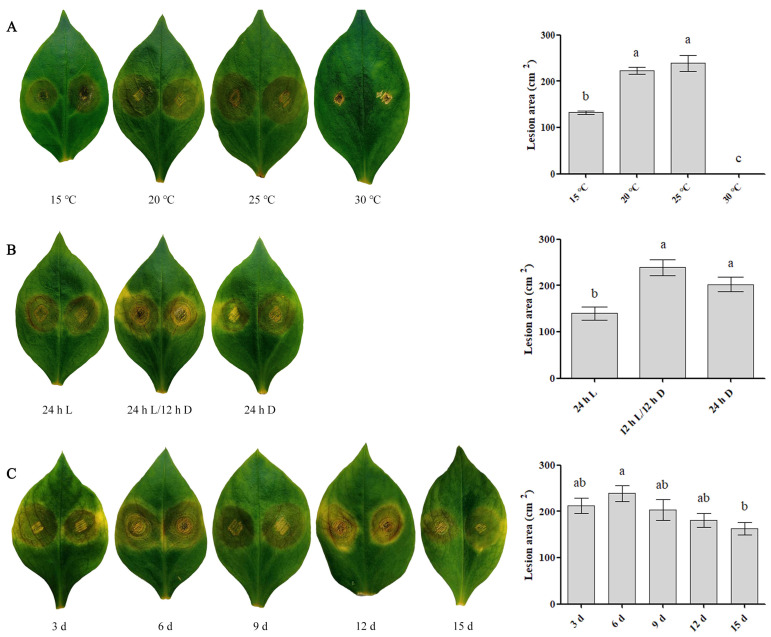
Effects of environmental conditions and mycelial age on infection in *P. heterophylla* by *S. versabilis*. (**A**) Temperature treatment. (**B**) Light regime. (**C**) Mycelial age. Lesion areas were measured at 7 days post inoculation. Data represent means ± SE of three independent biological experiments, each with three technical replicates. Different lowercase letters indicate significant differences (*p* < 0.05) as determined by One-way ANOVA followed by Tukey’s multiple comparison test using SPSS 19.0. 24 h L = continuous light; 12 h L/12 h D = 12 h light/12 h darkness; 24 h D = continuous darkness.

Across all treatments, visible symptoms of *S. versabilis* infection on detached injured leaves of *P. heterophylla* consistently appeared at approximately 3 d post inoculation. Under a 12 h L/12 h D photoperiod, successful infections were established at temperatures ranging from 15 °C to 25 °C. Lesion development differed markedly among temperature treatments, with significantly larger lesion areas recorded at 20 °C and 25 °C after 7 d (*p* < 0.05), and maximal lesion expansion observed at 25 °C (Figure 7A).

Light conditions also significantly influenced infection severity. At 25 °C, leaves incubated under a 12 h L/12 h D cycle or continuous darkness developed significantly larger lesions than those under continuous light (*p* < 0.05), with the greatest lesion expansion occurring under a 12 h L/12 h D cycle (Figure 7B).

Mycelial age further affected pathogenicity. Inoculation with 3–12 d-old cultures resulted in consistent lesion formation (Figure 7C), indicating maximal infectivity within this developmental window.

In general, optimal infection was observed when wounded leaves were inoculated with 3–12 d-old mycelial plugs and incubated at 25 °C under either a 12 h L/12 h D photoperiod or continuous darkness.

### 3.5. Cytology of the Infection Process of S. versabilis in P. heterophylla

Following inoculation onto onion epidermis, conidia germinated and produced germ tubes by 6 hpi (Supplementary Figure S5A). Germ tubes elongated into hyphae, and swelling of cells adjacent to the conidia became evident l between 12 and 24 hpi (Supplementary Figure S5B,C). Hyphae penetrated the epidermis directly and increased in diameter immediately after penetration, followed by continuous extension. No specialized infection structures were observed by 36 hpi (Supplementary Figure S5D,E). By 48 hpi, invasive hyphae had branched extensively and spread throughout the epidermal cell layers (Supplementary Figure S5F).

**Figure 8:**
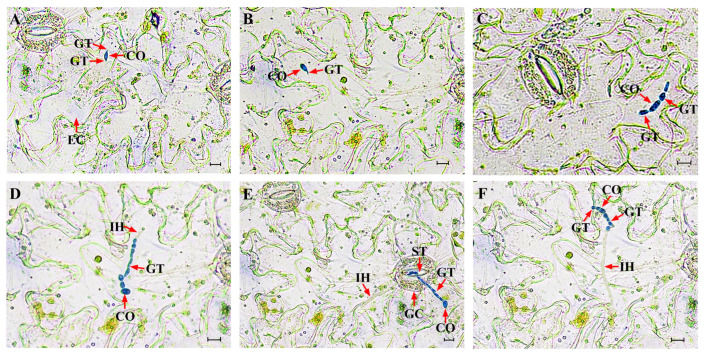
Cytological observations of the infection process of *S. versabilis* on *P. heterophylla* leaves. (**A**,**B**) Conidia germinated with one to two germ tubes at 6–12 hours post-inoculation (hpi). (**C**) Germ tubes elongated into hyphae, and localized swelling of cells adjacent to the conidia by 24 hpi. (**D**,**E**) Early infection events at 48–60 hpi ((**D**). Hyphae penetrated epidermal cells directly. (**E**). Hyphae entered into host tissues through the open stomata). (**F**) By 72 hpi, the invasive hyphae had further colonized host cells. CO = conidium. GT = germ tube. EC = epidermal cell. IH = invasive hyphae. GC= guard cell. ST = stoma. Scale Bar = 10 μm.

A similar infection sequence was observed on *P. heterophylla* leaves, although progression was slightly slower than that on onion epidermis. Conidia germinated and produced one to two germ tubes at 6–12 hpi (Figure 8A,B). Germ tubes subsequently elongated into hyphae, and localized swelling of cells adjacent to the conidia was detected at 24–36 hpi (Figure 8C). Invasive hyphae formed immediately after penetration of the leaf cuticle and no specialized penetration structures were observed. Some hyphae penetrated directly through the cuticle, whereas others entered through stomata or intercellular spaces. At 48–60 hpi, most hyphae penetrated primarily via the intercellular spaces (Figure 8D,E). By 72 hpi, invasive hyphae had advanced into neighboring cells, indicating active colonization, indicating active colonization (Figure 8F).

## 4. Discussion

### 4.1. Taxonomic Resolution and Clarification of Conflicting Reports

Accurate identification of plant pathogenic fungi is fundamental for understanding disease epidemiology and developing effective control strategies, particularly for medicinal crops that are highly sensitive to foliar damage and chemical residues. During the past decade, extensive taxonomic revisions within *Didymellaceae* have reshaped the classification of phoma-like fungi based on multi-locus phylogenetic analyses combined with detailed morphological characteristics, enabling the resolution of cryptic species complexes and improving diagnostic reliability [22,23,24,42]. Within this revised framework, *Ascochyta versabilis*, historically classified as *Phoma versabilis* [43], was transferred to *Sclerotiophoma versabilis* based on multi-locus phylogenetic evidence [24].

In this study, the causal agent of leaf spot disease in *P. heterophylla* was conclusively identified as *S. versabilis* through the integration of detailed morphology and a multi-locus (ITS-LSU-*rpb2*-and-*tub2*) phylogenetic analysis. This result aligns with recent reports based on molecular phylogenetic analyses [11,14], while providing stronger evidentiary support than earlier studies that relied primarily on limited morphological traits or on restricted molecular markers. Notably, the conidial morphology described by Yao et al. [14] differs from both Li et al. [11] and the present observations, raising concerns regarding the reliability of their identification.

Earlier attributions of the disease to *Phyllosticta commonsii* [10,19] or broader phoma-like taxa [17,18] were largely based on morphological observations alone and lacked molecular validation. Such discrepancies likely reflect both the historical instability of phoma-like taxonomy and the limited resolving power of morphology alone when species boundaries are cryptic. By integrating multi-locus phylogenetic inference with comprehensive cultural and micromorphological characterization, the present study establishes a robust taxonomic framework that provides a reliable foundation for future epidemiological investigations and disease management strategies in this pathosystem.

### 4.2. Environmental Drivers and Implications for Disease Management

Beyond taxonomic confirmation, our study advances understanding of the ecological drivers of leaf spot epidemics in *Pseudostellaria heterophylla* by defining quantitative environmental thresholds governing mycelial growth, conidial germination, and infection. *S. versabilis* exhibited maximal mycelial growth and conidial germination at moderate temperatures (approximately 20 to 25 °C) under weakly acidic to neutral conditions, with pH 6–8 for vegetative growth and pH 6–7 for germination. These processes were further enhanced by continuous illumination, whereas infection efficiency peaked at 20 to 25 °C under either a 12 h L/12 h D cycle or continuous darkness.

These physiological optima correspond closely to climatic conditions prevailing during the main disease outbreak periods in major *P. heterophylla* production regions in China, which are characterized by mild temperatures and high humidity during spring and early summer [12]. Such conditions promote prolonged leaf wetness and favorable canopy microclimates, which are well-recognized prerequisites for successful infection and polycyclic epidemic development in many *Didymellaceae*-associated diseases [44].

Accordingly, our laboratory findings are consistent with a temperature- and moisture-driven epidemiological framework for leaf spot development in *P. heterophylla*. Under dense planting and continuous monoculture systems, even modest shifts toward these optimal environmental conditions may substantially increase effective inoculum pressure, conidial germination, and penetration success by prolonging leaf wetness and creating a more favorable canopy microclimate [44,45,46,47]. Moreover, the strong inhibition of growth and infection at elevated temperatures offers a mechanistic explanation for the seasonal decline in disease pressure during hotter periods.

Collectively, these experimentally defined environmental limits provide a mechanistic basis for disease-risk assessment and contribute to a better understanding of epidemic dynamics [48].

### 4.3. Nutritional Plasticity and Infection Strategy

Nutritional profiling revealed *S. versabilis* efficiently utilizes diverse carbon and nitrogen sources, with starch, lactose, glucose, glycine, and peptone supporting particularly vigorous growth. This broad metabolic capacity reflects pronounced physiological plasticity, which likely facilitates persistence under fluctuating environmental and nutritional conditions.

Such nutritional versatility is commonly associated with necrotrophic and facultative necrotrophic pathogens, which rely on rapid exploitation of host-derived substrates released during tissue senescence and lesion expansion [49,50,51]. In contrast to obligate biotrophs, these pathogens possess wide substrate utilization capacities that enable sustained colonization of damaged or aging tissues.

Cytological observations further elucidated the infection strategy of *S. versabilis*. The pathogen penetrated host tissue either directly or via stomata without forming specialized infection structures such as appressoria or haustoria. Similar penetration strategies have been reported in several *Didymellaceae* species and other plant pathogens, including *Fusarium oxysporum* f. sp. *vasinfectum* [52,53,54]. Direct penetration in the absence of appressoria typically requires coordinated secretion of cell-wall-degrading enzymes and phytotoxic compounds [55,56].

The absence of haustorial interfaces suggests that sustained maintenance of living host cells is not required for nutrient acquisition. Instead, rapid hyphal proliferation and progressive lesion development are consistent with a strategy centered on host cell disruption and nutrient liberation, characteristic of necrotrophic or hemibiotrophic fungi [51,57]. Well-characterized necrotrophs, including *Alternaria alternata*, *Botrytis cinerea*, and *Sclerotinia sclerotiorum*, exhibit similar physiological and biochemical traits [49,50,58]. In agreement with this pattern, previous studies have reported the production of phytotoxic compounds by *S. versabilis* [59], providing additional biochemical support for a necrotrophic-leaning ecological strategy.

Taken together, the physiological, cytological, and biochemical evidence suggests that this pathogen primarily exhibits functional traits consistent with necrotrophic lifestyles. Nevertheless, definitive classification will require future molecular analyses of toxin biosynthesis, cell-wall-degrading enzymes, and effector repertoires.

### 4.4. Implications for Integrated Disease Management and Forecasting

Recent studies emphasize that plant–fungal interactions are highly dynamic processes governed by complex molecular, physiological, and ecological adjustments on both sides [60,61,62,63]. Building upon the ecological and biological insights obtained here, integration of environmental thresholds, nutritional requirements, and infection biology provides a scientifically grounded basis for improving leaf spot management in commercial *P. heterophylla* production systems.

The experimentally defined optima for growth, germination, and infection may be translated into practical infection-risk indicators, thereby facilitating a shift from calendar-based fungicide applications to predictive, risk-based interventions [64]. Furthermore, the demonstrated infection routes highlight the central role of canopy microclimate in epidemic development, particularly leaf wetness duration and relative humidity. Cultural practices that reduce canopy density, improve air circulation, optimize planting spacing, and minimize mechanical injury are therefore expected to represent effective, low-resistance-risk control measures [65].

Given the increasing constraints on chemical fungicide use arising from resistance development and environmental concerns, durable disease control requires integrating reduced-risk fungicides with nonchemical approaches, including cultural regulation, resistant germplasm deployment, and biological control. In this context, the present study provides a scientific basis for optimizing spray timing, minimizing selection pressure, and supporting long-term sustainable disease management strategies [66].

### 4.5. Limitations and Future Perspectives

Despite providing an integrated framework linking taxonomy, physiology, and infection cytology, several limitations should be acknowledged. First, environmental thresholds were primarily derived from controlled laboratory assays using detached leaves; field validation across multiple regions and seasons is required to strengthen predictive applicability. Second, although cytological observations clarified penetration routes, the molecular basis of penetration and tissue necrosis remains unresolved. Future studies should characterize secreted enzymes, secondary metabolites, and gene expression dynamics during early infection. Third, population-level variation among geographically distinct isolates was not addressed and warrants further investigation to assess adaptive potential and epidemiological diversity.

## 5. Conclusions

This study provides a comprehensive identification of *S. versabilis* as the causal agent of leaf spot disease in *P. heterophylla* through integrated morphological characterization and multi-locus phylogenetic analyses. By combining physiological assays, infection experiments, and cytological observations, we present the first systematic elucidation of the infection biology and quantitative environmental thresholds of this pathogen. The results demonstrated that the pathogen grew, germinated and infected optimally at moderate temperatures (20–25 °C), weakly acidic to neutral pH (6–7), and appropriate light conditions favor fungal growth, germination, and infection. Nutritional profiling further revealed a broad metabolic capacity, reflecting pronounced physiological plasticity. In addition, cytological analysis showed that *S. versabilis* exhibits a direct penetration strategy without the formation of specialized infection structures such as appressoria, suggesting a necrotrophic-leaning infection behavior. Taken together, these findings clarify the taxonomic status and ecological drivers of leaf spot development in *P. heterophylla*, and provide a biologically informed basis for disease-risk assessment and integrated management. Future studies incorporating field-based validation, population-level analyses, and molecular characterization of pathogenicity factors will further strengthen understanding of epidemic dynamics in this pathosystem.

## Figures and Tables

**Table 1:** Primers used in molecular phylogenetic analyses, with originating loci, PCR amplification procedures with minor modifications, and references.

Gene/DNA Regions	Primers		PCR Amplification Procedures
	Name	Sequence (5′ → 3′)	
ITS	ITS1	TCCGTAGGTGAACCTGCGG	95 °C 5 min; 32 cycles of 95 °C 30 s, 48 °C 30 s, 72 °C 40 s; 72 °C 10 min; 10 °C soak
	ITS4	TCCTCCGCTTATTGATATGC	
LSU	LR0R	GTACCCGCTGAACTTAAGC	95 °C 5 min; 32 cycles of 95 °C 30 s, 48 °C 30 s, 72 °C 2 min; 72 °C 10 min; 10 °C soak
	LR7	TACTACCACCAAGATCT	
*rpb2*	RPB2-5F2	GGGGWGAYCAGAAGAAGGC	95 °C 5 min; 32 cycles of 95 °C 30 s, 54 °C 30 s, 72 °C 1 min; 72 °C 10 min; 10 °C soak
	fRPB2-7cR	CCCATRGCTTGYTTRCCCAT	
*tub2*	Btub2Fd	GTBCACCTYCARACCGGYCARTG	95 °C 5 min; 32 cycles of 95 °C 30 s, 52 °C 30 s, 72 °C 30 s; 72 °C 10 min; 10 °C soak
	Btub4Rd	CCRGAYTGRCCRAARACRAAGTTGTC	

ITS: internal transcribed spacer regions 1 and 2 including 5.8S nrDNA; LSU: 28S large subunit of the nrDNA; *rpb2*: partial RNA polymerase II second largest subunit gene; *tub2*: partial ß-tubulin gene.

**Table 2:** Information of the species used in this study.

Species	Strain Number	Status	Host, Substrate	Country	GenBank Accession Numbers3			
					ITS	LSU	*rpb2*	*tub2*
*Didymella ocimicola*	LC 8138		*Ocimum* sp.	China	KY742079	KY742233	MT018180	KY742321
*Ectodidymella nigrificans*	CBS 100190	R	*Brassica napus*	Germany	GU237708	GU237967	MT018078	GU237530
*Ectophoma insulana*	CBS 252.92	T	*Olea europaea*	Greece	MN973481	MN943685	MT018070	MT005581
*Epicoccum poae*	CGMCC 3.18363	T	*Poa annua*	USA	KY742113	KY742267	KY742182	KY742355
*Longididymella clematidis*	CBS 123705	T	*Clematis ligusticifolia*	USA	FJ515593	FJ515634	MT018076	FJ515611
*Pseudopeyronellaea eucalypti*	CPC 27678; CBS 142522	T	*Eucalyptus pellita*	Malaysia	KY979755	KY979810	KY979848	KY979921
*Remotididymella bauhiniae*	CBS 993.95	T	Soil in tropical forest	Papua New Guinea	MN973476	MN943679	MT018064	MT005576
*Sclerotiophoma versabilis*	CBS 876.97	R	*Silene* sp.	The Netherlands	GU237909	GU238152	MT018124	GU237664
*S. versabilis*	CBS 124689		Human toenail	Denmark	MN973517	MN943723	MT018123	MT005617
*S. versabilis*	KC1		*Pseudostellaria heterophylla* (Miq.) Pax	China	**PP683390**	**PP683405**	**PP693949**	**PP695666**
*Similiphoma crystallifera*	CBS 193.82	T	*Chamaespartium sagittale*	Austria	GU237797	GU238060	LT623267	GU237598

R: representative; T: ex-type. Newly generated sequences are indicated in bold.

## Data Availability

The original contributions presented in this study are included in the article/Supplementary Material. Further inquiries can be directed to the corresponding authors.

## Supplementary Materials

The following supporting information can be downloaded at: https://www.mdpi.com/article/10.3390/plants15060883/s1.
