# Supplementary material for: Identification and Biological Characterizations of the Causal Agent of Leaf Spot Disease in Pseudostellaria heterophylla
Source: Plants (Basel). 2026 Mar 12;15(6):883. doi: 10.3390/plants15060883 (PMC13029421; doi:10.3390/plants15060883)
Supplement: Supplementary file 1 [file plants-15-00883-s001.zip › plants-4131239-supplementary.pdf]

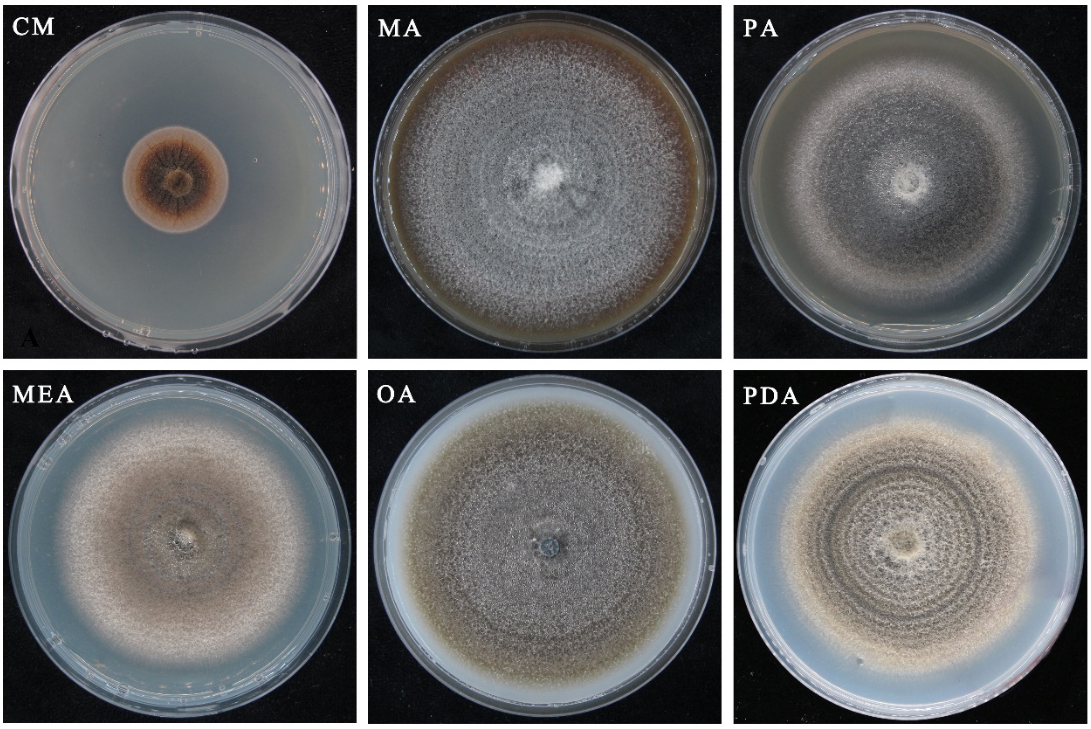

Figure. S1. Effects of media on mycelial growth of *S. versabilis*.

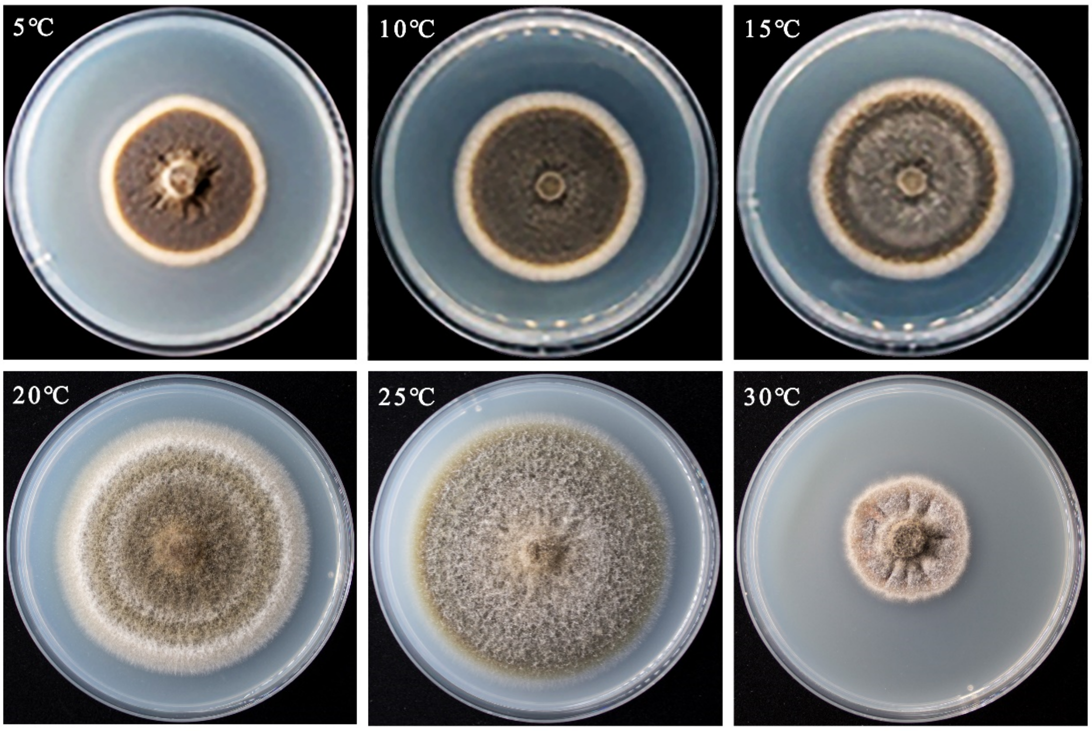

Figure. S2. Effects of temperatures on mycelial growth of *S. versabilis*.

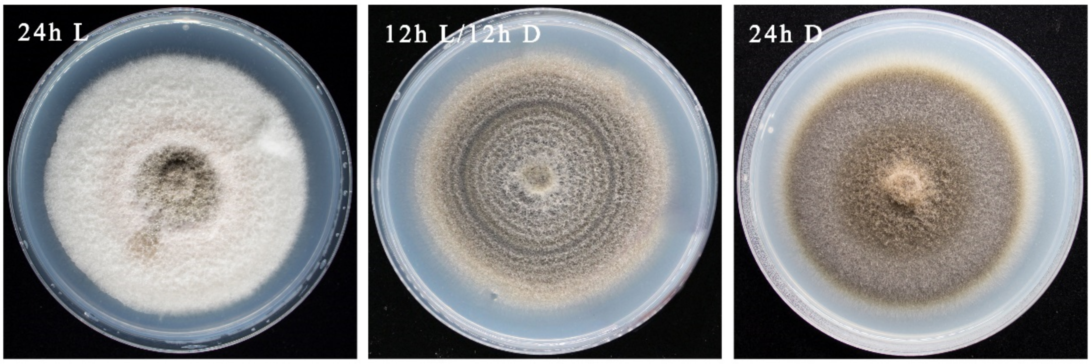

Figure. S3. Effects of light regimes on mycelial growth of *S. versabilis*.

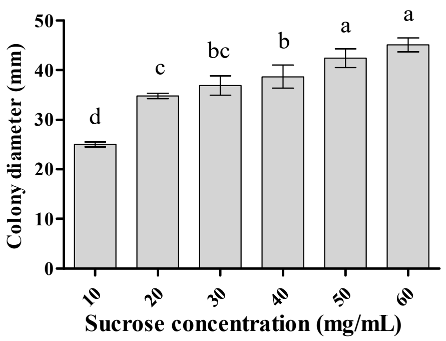

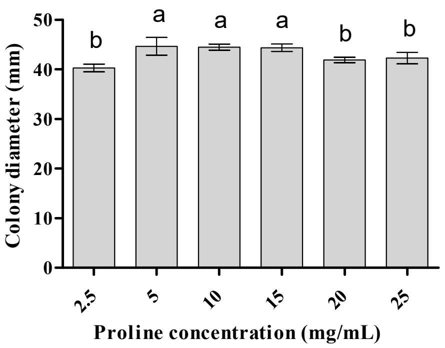

A **B**

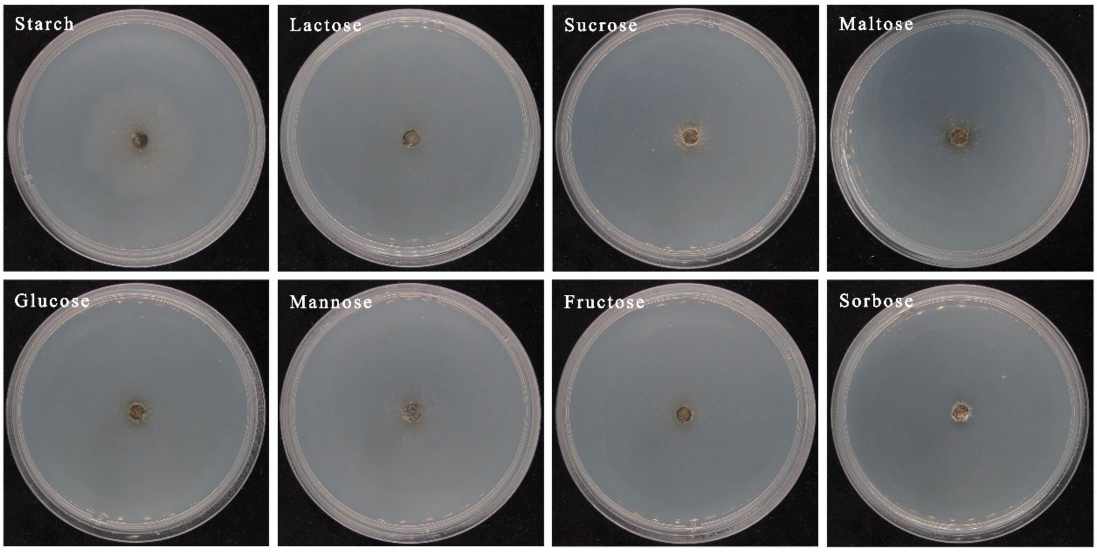

C

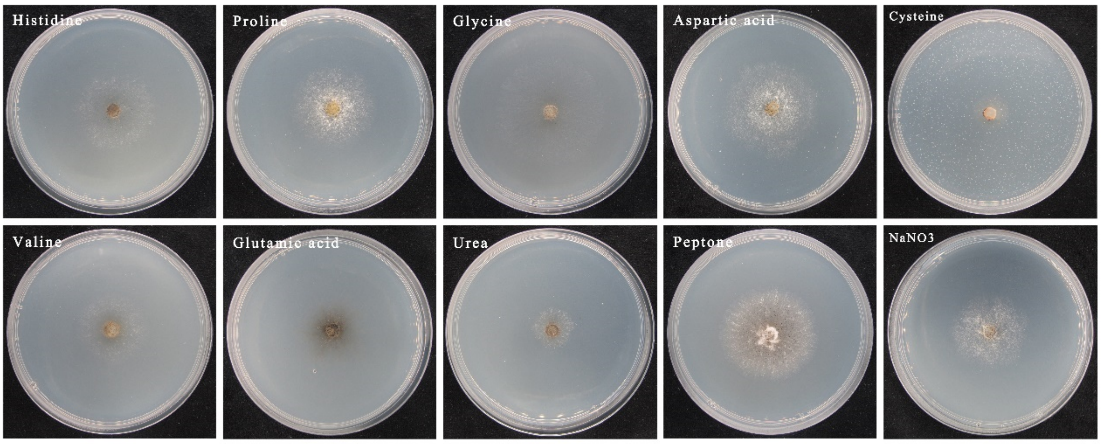

D

Figure S4. Effects of carbon and nitrogen sources on mycelial growth of *S. versabilis*. (A-B) Sucrose and proline were used to determine the appropriate concentrations for the carbon and nitrogen source assays, respectively. (**C**) Effects of different carbon sources. (**D**) Effects of different nitrogen sources. Data represent means ± SE of three independent biological experiments, each with three technical replicates. Different superscript letters indicate significant differences ($p < 0.05$) as determined by One-way ANOVA followed by Turkey's multiple comparison test in SPSS 19.0.

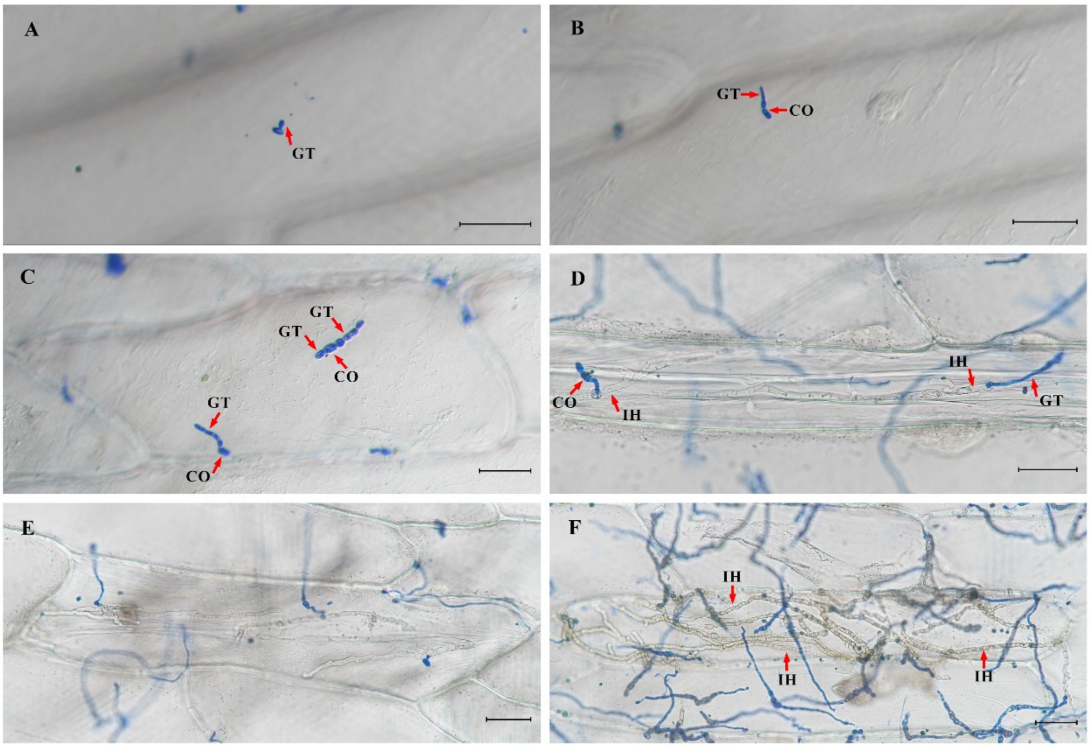

Figure S5. Cytological observations of *S. versabilis* infection on onion epidermis. (**A**) Conidia germinated and produced germ tubes by 6 hpi. (**B–C**) Germ tubes elongated into hyphae, cells adjacent to the conidia began to swell at 12–24 hpi. (**D–E**) Hyphae penetrated epidermal cells directly and initiated infection by 36 hpi. (**F**) Invasive hyphae branched extensively and further colonized host tissues by 48 hpi. hpi=hours post-inoculation. CO=conidium. GT=germ tube. IH=invasive hyphae. Scale bar = 50 μm.
